# Use, perceptions, and effectiveness of e-cigarettes for smoking cessation among older adults in England: a population study, 2014–2024

**DOI:** 10.1186/s12916-024-03728-x

**Published:** 2024-10-31

**Authors:** Sarah E. Jackson, Jamie Brown, Lion Shahab, Sharon Cox

**Affiliations:** 1 Department of Behavioural Science and Health, University College London, 1-19, London, Torrington Place WC1E 7HB UK; 2 SPECTRUM Consortium, Edinburgh, UK

**Keywords:** Elderly, Ageing, Old age, Tobacco, Cigarettes, Vaping

## Abstract

**Background:**

This study aimed to characterise patterns of tobacco smoking and vaping among older adults (≥ 65 years) in England, to explore harm perceptions of e-cigarettes among those who smoke, and to estimate the real-world effectiveness of e-cigarettes for helping older adults to stop smoking.

**Methods:**

Data were collected as part of a representative monthly cross-sectional household survey in England between April 2014 and April 2024 (*n* = 197,219). We analysed differences between older (≥ 65 years) and younger/middle-aged adults (18–64 years) in (a) time trends in tobacco smoking and vaping, (b) harm perceptions of e-cigarettes vs. cigarettes (adjusting for gender, socioeconomic position, and vaping status), and (c) the real-world effectiveness of e-cigarettes for smoking cessation (adjusting for gender, socioeconomic position, characteristics of the quit attempt, and use of other evidence-based cessation aids).

**Results:**

Tobacco smoking prevalence remained relatively unchanged over time among older adults (at ~ 9%; 9.5% [8.5–10.6%] in April 2014 and 8.7% [7.7–9.8%] in April 2024) but vaping prevalence increased (from 2.1% [1.6–2.7%] to 3.7% [3.0–4.6%], respectively). These trends differed from those observed among younger/middle-aged adults, among whom there was a clear decline in smoking (from 21.8% [21.0–22.7%] to 18.2% [17.3–19.0%]) and a larger increase in vaping (from 5.6% [5.2–6.1%] to 16.2% [15.3–17.0%]). Older adults were consistently less likely than younger/middle-aged adults to use e-cigarettes to support attempts to quit smoking (26.8% [17.2–39.3%] vs. 43.7% [39.6–48.0%] in April 2024). Older smokers reported greater uncertainty about the harms of e-cigarettes compared with cigarettes (OR_adj_ = 2.48 [2.28–2.69]). E-cigarettes appeared to be effective for helping older adults to stop smoking (OR_adj_ = 1.50 [0.96–2.34]); whether effectiveness was lower than for younger/middle-aged adults was inconclusive.

**Conclusions:**

Over the past decade, smoking prevalence has remained stable among older adults while decreasing among the rest of the adult population in England. Older adults are more unsure about the relative harms of e-cigarettes and less likely to use them to support attempts to quit smoking, despite evidence that they are effective for smoking cessation in this population.

**Supplementary Information:**

The online version contains supplementary material available at 10.1186/s12916-024-03728-x.

## Background

Reducing tobacco smoking prevalence remains a global public health priority. It can be achieved by reducing rates of initiation and increasing rates of cessation among people who smoke. Stopping smoking is associated with substantial improvements in health and life expectancy at any age [1–5]. In the context of an ageing population, it is particularly important to reduce tobacco smoking rates among older adults as they make up an increasing proportion of the population, and smoking exacerbates age-related illnesses [6, 7]. In the UK, 8.3% of older adults (≥ 65 years) smoke cigarettes [8]; just over 1 million people [9]. Use of e-cigarettes (vaping) can play a role in helping people stop smoking: evidence suggests e-cigarettes are both more effective than nicotine replacement therapy (NRT)[10, 11] and much less harmful than cigarettes [12]. However, older adults show a preference for NRT [13]. Research on smoking and vaping has largely focused on younger age groups or the entire adult population. Understanding patterns of tobacco smoking, and perceptions and use of e-cigarettes, among older adults can inform the development of interventions to boost quitting in later life.

Data from national surveys suggest relative declines in tobacco smoking have been slower among older (≥ 65 years) than younger adults in recent years [8, 14]. In the USA, a recent study showed that while there had been dramatic declines in adult smoking prevalence over the last decade (2011–2022), there was no decrease among older adults. In fact, there was an uncertain *increase* in smoking rates among older adults from lower income brackets (< 200% of the federal poverty level), from 13.0% (95% CI 11.2–14.7%) in 2011 to 15.8% (14.1–17.6%), and no notable change among those on higher incomes [14]. In the UK, the Annual Population Survey also suggests declines in smoking have been less pronounced among older adults [8]. Between 2011 and 2022, cigarette smoking rates fell by just 1.8 percentage points (a relative decline of 17.8%) among those aged ≥ 65 [8], compared with 4.4 (24.4%), 6.7 (31.9%), 8.0 (35.6%), 8.7 (34.8%), and 13.4 (53.6%) among those aged 55–64, 45–54, 35–44, 25–34, and 18–24, respectively [8]. These estimates do not take into account people who only smoke non-cigarette tobacco (e.g. cigarillos, cigars, shisha), which has increased in England in recent years[15, 16] – particularly among younger adults [15], so they likely overestimate declines in total tobacco smoking prevalence.

At the same time, uptake of vaping has also been lower among older adults than in younger age groups [17]. E-cigarettes became popular in England between 2011 and 2013, and up until 2020, there was not a clear age gradient in vaping: prevalence was consistently lowest among those aged ≥ 65 and was higher (and somewhat similar) among those aged 16–24, 25–34, 35–44, 45–54, and 55–64 [17]. In the last few years, vaping prevalence has increased rapidly among adults in England [17, 18]. This increase has followed an inverse age gradient, with the greatest increases at younger ages [17, 18].

While e-cigarettes are the smoking cessation aid most commonly used by adults in England overall (used in more than one in every three quit attempts) [17], and have been since 2013 [17], they appear to be used less often for this purpose by older than younger smokers (at least up to 2018) [13]. It is not clear why this is: it could be that older adults are slower to adopt new technology, or it is possible that older adults may have less favourable perceptions of vaping versus smoking compared with those who are younger (e.g. because they are more concerned about potential harms to health or see e-cigarettes as products intended for use by younger people). The accuracy of harm perceptions of e-cigarettes among smokers in England has deteriorated over recent years [19]; how they have changed among older adults specifically is not clear.

E-cigarettes have also been found to be one of the most effective methods for stopping smoking [20]. Estimates suggest they have helped in the region of 30,000 to 50,000 additional smokers in England to quit each year since they have become popular, over and above the number who would otherwise have quit with other methods [21]. To our knowledge, their effectiveness has not been examined among older smokers specifically. If older adults are less likely to use e-cigarettes as a route to quit smoking, or if e-cigarettes are less effective when used by older smokers, this may be contributing to slower declines in smoking prevalence in this demographic.

This study aimed to characterise patterns of tobacco smoking and e-cigarette use (overall and to support attempts to quit smoking) over the past decade among older adults (≥ 65 years) in England. It also aimed to explore harm perceptions of e-cigarettes among those who smoke and to estimate the real-world effectiveness of e-cigarettes for helping older adults to stop smoking. We also provided corresponding data for younger and middle-aged adults (18–64 years), to contextualise our findings.

## Methods

### Pre-registration

The study protocol, research questions, and analysis plan were pre-registered on Open Science Framework (https://osf.io/yg7nr/). We made one amendment to a sensitivity analysis of the real-world effectiveness of e-cigarettes for smoking cessation following peer review. This was to adjust for whether the participant had received advice on smoking from GP in the past year, rather than simply whether they had visited their GP in the past year, for a more direct assessment of potential to have received advice on e-cigarettes. We also added an unplanned analysis of age-specific changes in harm perceptions of e-cigarettes compared with cigarettes across the study period.

### Design

We analysed data from the Smoking Toolkit Study, an ongoing monthly cross-sectional survey of a nationally representative sample of adults in England [22]. The study uses a hybrid of random probability and simple quota sampling to select a new sample of ~ 1700 adults each month. Interviews are held with one household member in selected geographic output areas until quotas are fulfilled. The quotas are based on factors influencing the probability of being at home (i.e. working status, age and gender). Full details of the sampling procedure are provided elsewhere [22, 23].

Data were collected monthly through face-to-face computer-assisted interviews up to February 2020. However, due to social distancing restrictions under the COVID-19 pandemic, no data were collected in March 2020 and since April 2020 data have been collected via telephone. The telephone-based data collection relies upon a similar combination of random location and quota sampling, and weighting approach as the face-to-face interviews. The two data collection modalities show good comparability: since restrictions were lifted, we have run parallel waves of face-to-face and telephone data collection, which yielded similar estimates of key smoking and nicotine use variables [24]. It is not possible to compare response rates because the hybrid sampling approach allows interviews to select households within randomly selected geographic areas that are most likely to fulfil their quotas. While in theory, it is possible for a participant to be included in more than one wave, this is very unlikely given the numbers sampled (we cannot determine whether any such cases exist because all data are fully anonymised).

For the present study, we used data from respondents to the survey over a 10-year period from April 2014 to April 2024 (the most recent data available at the time of analysis). Our primary focus was older adults (which we defined as those aged ≥ 65 years [25]). We also included data from younger and middle-aged adults (18–64 years) in England to examine the extent to which the patterns of results we observed were unique to older adults.

### Measures

#### Smoking status and quitting

Tobacco smoking status was assessed by asking participants which of the following best applied to them: (a) I smoke cigarettes (including hand-rolled) every day; (b) I smoke cigarettes (including hand-rolled), but not every day; (c) I do not smoke cigarettes at all, but I do smoke tobacco of some kind (e.g. pipe, cigar or shisha); (d) I have stopped smoking completely in the last year; (e) I stopped smoking completely more than a year ago; (f) I have never been a smoker (i.e. smoked for a year or more). Those who responded a–c were considered current smokers, those who responded d–e ex-smokers, and those who responded f never-smokers. Those who responded a-d were considered past-year smokers. Note that our definition of current smoking includes people who exclusively smoke non-cigarette tobacco products, so estimates of smoking prevalence differ from other surveys in England that focus specifically on cigarette smoking (e.g. the Annual Population Survey [8]).

Quit attempts were assessed among past-year smokers with the question: ‘How many serious attempts to stop smoking have you made in the last 12 months? By serious attempt I mean you decided that you would try to make sure you never smoked again. Please include any attempt that you are currently making and please include any successful attempt made within the last year’.

Short-term (< 1 year) success of quit attempts was assessed among past-year smokers who made at least one attempt to quit in the past year with the question: ‘How long did your most recent serious quit attempt last before you went back to smoking?’ Those who reported that they were still not smoking (i.e. were continuously abstinent from the start of the most recent attempt up to the time of the survey) were coded 1, else they were coded 0.

#### E-cigarette use and perceptions

Current vaping was assessed within several questions that asked participants about the use of a range of nicotine products. Current smokers were asked ‘Do you regularly use any of the following in situations when you are not allowed to smoke?’ and those who reported cutting down ‘Which, if any, of the following are you currently using to help you cut down the amount you smoke?’; past-year smokers were asked ‘Can I check, are you using any of the following either to help you stop smoking, to help you cut down or for any other reason at all?’; and non-smokers were asked ‘Can I check, are you using any of the following?’. Those who reported using an e-cigarette in response to any of these questions were considered current vapers (coded 1), else they were considered non-vapers (coded 0).

Use of e-cigarettes in quit attempts was assessed among past-year smokers who made at least one attempt to quit in the past year with the question: ‘Which, if any, of the following did you try to help you stop smoking during the most recent serious quit attempt?’. Participants were asked to indicate all that apply. Those who responded ‘electronic cigarette’ were coded 1, else they were coded 0. Use of other evidence-based cessation aids was controlled for in the analyses (see covariates). We did not consider previous failed quit attempts with vaping to avoid potential confounding by the number of past-year quit attempts.

Harm perceptions of e-cigarettes were assessed among current smokers with the question: ‘Compared to regular cigarettes, do you think electronic cigarettes are more, less, or equally harmful to health?’ Response options were ‘more harmful’, ‘less harmful’, ‘equally harmful’, or ‘don’t know’. We dummy coded these response options as one-versus-else (e.g. less harmful vs. all other responses) for analysis [19]. This variable was first included in the survey in November 2014, so analyses of this outcome were restricted to data collected from this wave onwards.

#### Occupational social grade

Occupational social grade was defined according to the National Readership Survey classification [26] and categorised as ABC1 (includes managerial, professional, and upper supervisory occupations) and C2DE (includes manual routine, semi-routine, lower supervisory, long-term unemployed, and state pension). Retired people who have a company pension or private pension, or who have private means, were graded on their previous occupations. If another job is taken after retirement, they were graded according to their highest occupation, as long as there was a pension or financial means derived from that occupation. Retired people who received a pension based on a job held by their late spouse or civil partner were graded based on that occupation. This occupational measure of social grade is a valid index of socioeconomic status, widely used in research in UK populations, which is particularly relevant in the context of tobacco use [27].

#### Covariates

Consistent with previous studies [13, 28], covariates for the real-world effectiveness analysis included gender, occupational social grade, level of cigarette addiction (indexed by a single-item measure of strength of urges to smoke [29]), time since the quit attempt started, the number of prior past-year quit attempts, whether the quit attempt was planned, whether the respondent cut down first, use of other evidence-based cessation aids (NRT/varenicline/bupropion/face-to-face behavioural support), and the month and year of survey.

We included other covariates in two sensitivity analyses. The first substituted the highest level of education for occupational social grade, as a measure of socioeconomic position that is less affected by retirement status. The second additionally adjusted for whether the participant received advice on smoking from their GP in the past year, given older adults are likely to have more contact with their GP and if they are either encouraged or discouraged to continue using e-cigarettes beyond a quit attempt through interactions with their GP, they may be more or less likely to continue using them and less or more likely to relapse to smoking.

### Statistical analysis

Data were analysed in R v.4.2.1. Missing cases were excluded on a per-analysis basis (< 5% across all variables). The Smoking Toolkit Study uses raking to weight the sample to match the population of England in terms of key demographics [22]. The following analyses used weighted data.

#### Time trends in smoking and vaping

Trend analyses used monthly data collected across the study period (April 2014 to April 2024).

We used logistic regression models to estimate monthly time trends in each outcome among older (≥ 65 years) compared with younger and middle-aged (18–64 years) adults. Models included time, age category, and their interaction as predictors—thus allowing for time trends to differ between older vs. younger and middle-aged adults. Time (survey wave) was modelled continuously using restricted cubic splines with five knots (placed at equal percentiles of the data), to allow relationships with time to be flexible and non-linear, while avoiding categorisation.

To explore differences in smoking and vaping trends by occupational social grade, we repeated these models testing the three-way interaction between time, age, and occupational social grade (ABC1/C2DE).

To explore differences in vaping trends by smoking status, we repeated this model testing the three-way interaction between time, age, and smoking status (current/ex/never smoker).

We used predicted estimates from our models to (i) plot the prevalence of each outcome over the study period (among older adults and among younger and middle-aged adults) and (ii) derive up-to-date estimates of the prevalence of each outcome in April 2024.

#### Harm perceptions of e-cigarettes compared with cigarettes

The harm perceptions analysis used data aggregated across survey waves. The primary analysis used data collected between November 2014 (the first wave to assess harm perceptions) and April 2024. A sensitivity analysis used data collected between April 2022 and April 2024 (the most recent 2 years of data) to offer insight into current harm perceptions, given there have been substantial changes in perceptions across the period [19]. We also provided annual descriptive data on harm perceptions by age group across the study period (unplanned analysis).

We used logistic regression to examine associations between each harm perception (less harmful, equally harmful, more harmful, don’t know—dummy coded) and age (≥ 65 vs. 18–64 years), with and without adjustment for gender, occupational social grade, and vaping status. We repeated this analysis stratified by vaping status, to explore differences between exclusive smokers and those who also used e-cigarettes (‘dual users’).

#### Real-world effectiveness of e-cigarettes for smoking cessation

The real-world effectiveness analysis used data aggregated across survey waves (April 2014 to April 2024).

Among past-year smokers who tried to quit in the past year, we used logistic regression to test whether the association between self-reported abstinence (abstinent yes vs. no) and use of e-cigarettes in the most recent quit attempt (yes vs. no) was moderated by age (≥ 65 vs. 18–64 years), using a two-way interaction. The model was adjusted for covariates as described in the measures section. We reran the model stratified by age group to provide more information as to the nature of any differences (or lack thereof). As sensitivity analyses, we repeated these analyses (i) adjusting for the highest level of education instead of occupational social grade and (ii) with additional adjustment for past-year GP contact.

In an unplanned post-hoc analysis, we calculated Bayes factors (BF) using an online calculator [30] to determine whether interaction results were supportive of the alternative hypothesis (i.e. the real-world effectiveness of e-cigarettes differs by age), the null hypothesis or were insensitive. We used a half-normal distribution, the mode at 0 (no effect), and the SD equal to the expected effect size, which we set at OR = 1.5 in either direction (i.e. OR = 1.5 indicating higher effectiveness among older adults and OR = 0.67 indicating lower effectiveness) [31]. BFs ≥ 3 can be interpreted as evidence for the alternative hypothesis (and against the null), BFs ≤ 1/3 as evidence for the null hypothesis, and BFs between 1/3 and 3 suggest the data are insensitive to distinguish the alternative hypothesis from the null [32, 33].

## Results

A total of 197,219 (unweighted) adults in England aged ≥ 18 years were surveyed between April 2014 and April 2024 (weighted mean [SD] age = 48.0 [18.6]; 50.8% [50.6–51.1%] women; 44.5% [44.3–44.7%] social grades C2DE). Of these, 145,681 (73.9%) were aged 18–64 years (mean [SD] age = 40.5 [13.4]; 49.8% [49.6–50.1%] women; 43.5% [43.2–43.8%] social grades C2DE) and 51,538 (26.1%) were aged ≥ 65 years (mean [SD] age = 74.1 [6.7]; 54.2% [53.8–54.7%] women; 47.9% [47.5–48.4%] social grades C2DE).

### Time trends in smoking and vaping

**Fig. 1:**
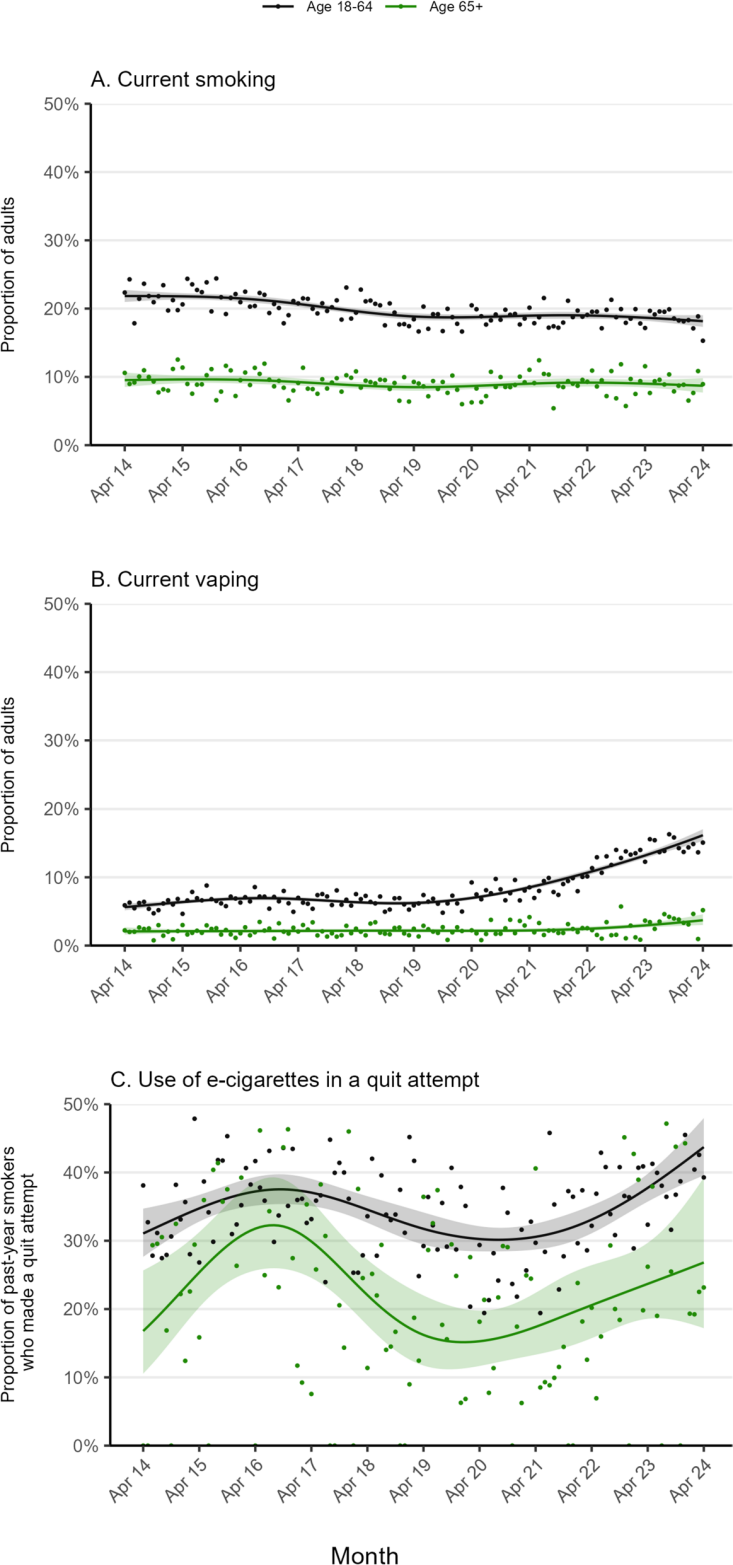
Trends in smoking and vaping among older (≥ 65 years) vs. middle-aged and younger adults (18–64 years) in England, April 2014 to April 2024. Panels show modelled time trends in **A** current smoking, **B** current vaping, and **C** use of e-cigarettes in quit attempts (defined as use in the most recent quit attempt among past-year smokers who made at least one serious attempt to quit in the past year). Lines represent the modelled weighted proportion by monthly survey wave (modelled non-linearly using restricted cubic splines with five knots). Shaded bands represent 95% confidence intervals. Points represent the unmodelled weighted proportion by month

**Fig. 2:**
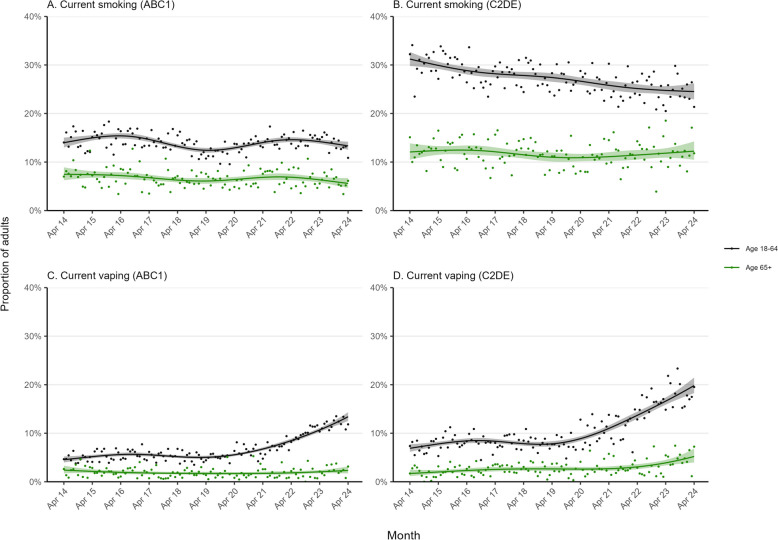
Trends in smoking and vaping among older (≥ 65 years) vs. middle-aged and younger adults (18–64 years) in England, by occupational social grade, April 2014 to April 2024. Panels show trends in the prevalence of **A**–**B** current smoking and **C**–**D** current vaping, by age and occupational social grade. ABC1 = more advantaged, C2DE = less advantaged. Lines represent the modelled weighted proportion by monthly survey wave (modelled non-linearly using restricted cubic splines with five knots). Shaded bands represent 95% confidence intervals. Points represent the unmodelled weighted proportion by month

Between April 2014 and April 2024, tobacco smoking prevalence decreased from 21.8% [21.0–22.7%] to 18.2% [17.3–19.0%] among younger and middle-aged adults but remained relatively unchanged among older adults (at around 9%; 9.5% [8.5–10.6%] in April 2014 and 8.7% [7.7–9.8%] in April 2024; Fig. 1A, Table 1). The decline among younger and middle-aged adults occurred predominantly among those from less advantaged social grades (C2DE), with little overall change among those from more advantaged social grades (ABC1; Fig. 2, Table 1). The opposite pattern was observed among older adults: no change among those from social grades C2DE but an uncertain decline among those from social grades ABC1 (Fig. 2, Table 1).

**Table 1:** Modelled estimates of changes in smoking and vaping outcomes among older adults (≥ 65 years) compared with younger and middle-aged adults (18–64 years) in England, from April 2014 to April 2024

	**Prevalence, % (95% CI)**^**a**^		
	***N***^**b**^	**April 2014**	**April 2024**
**Current smoking**			
Older adults	51,406	9.5 (8.5–10.6)	8.7 (7.7–9.8)
Younger/middle-aged adults	145,162	21.8 (21.0–22.7)	18.2 (17.3–19.0)
Social grades ABC1			
Older adults	29,526	7.5 (6.3–8.9)	5.6 (4.7–6.7)
Younger and middle-aged adults	90,229	14.0 (13.0–15.0)	13.3 (12.5–14.2)
Social grades C2DE			
Older adults	21,880	12.1 (10.5–13.9)	12.2 (10.5–14.3)
Younger and middle-aged adults	54,933	31.2 (29.8–32.7)	24.5 (22.9–26.2)
**Current vaping**			
Older adults	51,538	2.1 (1.6–2.7)	3.7 (3.0–4.6)
Younger/middle-aged adults	145,681	5.6 (5.2–6.1)	16.2 (15.3–17.0)
Social grades ABC1			
Older adults	29,599	2.4 (1.7–3.3)	2.3 (1.7–3.2)
Younger and middle-aged adults	90,559	4.5 (4.0–5.2)	13.4 (12.5–14.3)
Social grades C2DE			
Older adults	21,939	1.7 (1.2–2.5)	5.2 (4.0–6.8)
Younger and middle-aged adults	55,122	6.9 (6.2–7.7)	19.8 (18.3–21.5)
Never-smokers			
Older adults	29,753	0.1 (0.0–0.3)	0.1 (0.0–0.3)
Younger and middle-aged adults	91,019	0.2 (0.1–0.4)	5.0 (4.3–5.8)
Ex-smokers			
Older adults	16,901	1.6 (0.9–2.5)	6.7 (5.2–8.7)
Younger and middle-aged adults	25,832	8.3 (6.9–10.0)	30.4 (28.2–32.6)
Current smokers			
Older adults	4,752	15.9 (12.0–20.8)	15.0 (10.8–20.5)
Younger and middle-aged adults	28,311	19.9 (18.1–21.8)	36.1 (33.5–38.7)
**Use of e-cigarettes in a quit attempt**^**c**^			
Older adults	1,140	16.8 (10.5–25.6)	26.8 (17.2–39.3)
Younger/middle-aged adults	10,563	31.1 (27.6–34.7)	43.7 (39.6–48.0)

*CI*, confidence interval. ABC1 = more advantaged, C2DE = less advantaged

^a^Data for April 2014 and April 2024 are weighted estimates of prevalence in these months (the first and last in the study period) from logistic regression including an interaction between survey month modelled non-linearly using restricted cubic splines (five knots) and age group

^b^Unweighted sample size for each analysis. Note that there were some missing data on smoking status (unweighted *n* = 651, 0.3%); sample sizes show the number of participants contributing data to each analysis

^c^Among past-year smokers who made a past-year quit attempt

**Fig. 3:**
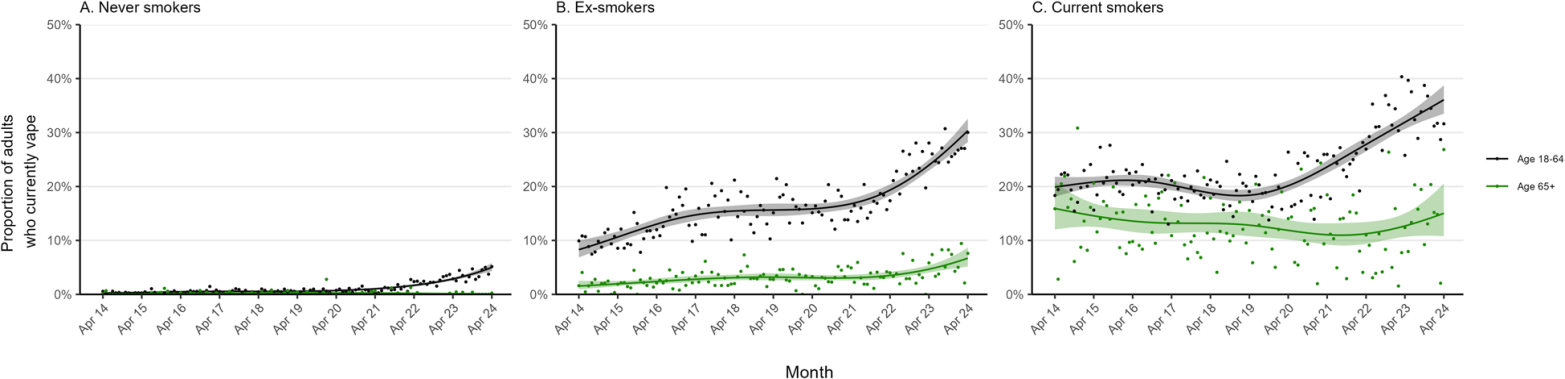
Trends in vaping among older (≥ 65 years) vs. middle-aged and younger adults (18–64 years) in England, by smoking status, April 2014 to April 2024. Panels show trends in the prevalence of current vaping, by age, among **A** never smokers, **B** ex-smokers, and **C** current smokers. Lines represent the modelled weighted proportion by monthly survey wave (modelled non-linearly using restricted cubic splines with five knots). Shaded bands represent 95% confidence intervals. Points represent the unmodelled weighted proportion by month

Vaping prevalence increased from 5.6% (5.2–6.1%) to 16.2% (15.3–17.0%) among younger and middle-aged adults and from 2.1% (1.6–2.7%) to 3.7% (3.0–4.6%) among older adults (Fig. 1B, Table 1). This increase occurred predominantly since early 2021. Among younger and middle-aged adults, the increase occurred across all social grades (Fig. 2, Table 1) and smoking statuses (Fig. 3, Table 1). However, among older adults, vaping only increased among social grades C2DE (Fig. 2, Table 1) and ex-smokers (Fig. 3, Table 1).

The prevalence of e-cigarette use in quit attempts increased from 31.1% (27.6–34.7%) to 43.7% (39.6–48.0%) among younger and middle-aged adults. There was also an uncertain increase among older adults, from 16.8% (10.5–25.6%) to 26.8% (17.2–39.3%) (Fig. 1C, Table 1). While the increase among older adults was uncertain, the relative percentage increase was larger for this group than for younger and middle-aged adults (59.5% vs. 40.5%). Changes were non-linear in both groups, increasing up to mid-2016, declining through to late-2019, and then increasing again up to April 2024. The initial increase and decline appeared more pronounced among older adults, although 95% CIs were wide.

### Harm perceptions of e-cigarettes compared with cigarettes

Among current smokers surveyed between November 2014 and April 2024 (unweighted *n* = 4439 older adults, *n* = 26,369 younger and middle-aged adults), misperceptions and uncertainty about the harms of e-cigarettes were common (Table 2). In both older and younger and middle-aged adults, only a minority of people believed e-cigarettes were less harmful (28.4% vs 34.8%, respectively). Older adults were much more likely to be uncertain about the harms, with 28.1% compared with 13.1% among younger and middle-aged adults responding that they did not know (OR_adj_ = 2.48 [2.28–2.69]). This greater percentage of uncertain older adults meant the absolute percentages in each of the other categories were lower than among younger and middle-aged adults, but the relative distribution of each perception was similar.

**Table 2:** Unadjusted and adjusted associations of age with harm perceptions of e-cigarettes relative to cigarettes, among current smokers

	**Prevalence, % (95% CI)**			
	**Middle-aged/younger adults**	**Older adults**	**OR (95% CI)**	**OR**_**adj**_** (95% CI)**
**All smokers (*n* = 30,808)**				
Less harmful	34.8 (34.2–35.4)	28.4 (27.0–29.8)	0.74 (0.69–0.80)	0.86 (0.79–0.92)
Equally harmful	37.2 (36.5–37.8)	32.6 (31.1–34.1)	0.82 (0.76–0.88)	0.76 (0.71–0.82)
More harmful	14.9 (14.4–15.4)	10.9 (9.9–11.9)	0.70 (0.62–0.78)	0.63 (0.57–0.70)
Don’t know	13.1 (12.7–13.6)	28.1 (26.6–29.5)	2.59 (2.38–2.81)	2.48 (2.28–2.69)
**Exclusive smokers (*n* = 24,213)**				
Less harmful	29.1 (28.4–29.7)	23.3 (21.9–24.7)	0.74 (0.68–0.81)	0.76 (0.70–0.83)
Equally harmful	39.3 (38.5–40.0)	34.3 (32.7–35.9)	0.81 (0.75–0.87)	0.79 (0.73–0.86)
More harmful	17.1 (16.5–17.7)	12.0 (10.9–13.1)	0.66 (0.59–0.74)	0.65 (0.58–0.73)
Don’t know	14.6 (14.0–15.1)	30.3 (28.8–31.9)	2.56 (2.35–2.78)	2.58 (2.37–2.82)
**Dual users (*n* = 6595)**				
Less harmful	53.9 (52.5–55.3)	63.0 (58.6–67.3)	1.45 (1.20–1.76)	1.55 (1.27–1.88)
Equally harmful	30.2 (29.0–31.5)	21.2 (17.6–24.9)	0.62 (0.50–0.78)	0.58 (0.46–0.73)
More harmful	7.6 (6.9–8.3)	3.1 (1.6–4.5)	0.39 (0.24–0.63)	0.38 (0.23–0.62)
Don’t know	8.3 (7.5–9.1)	12.7 (9.7–15.8)	1.61 (1.20–2.16)	1.62 (1.21–2.18)

*CI*, confidence interval

*OR*, odds ratio for harm perceptions among older adults (≥ 65 years) compared with younger and middle-aged adults (18–64 years; reference group)

*OR*_*adj*_, odds ratio adjusted for gender, occupational social grade, and (for analyses among all smokers) vaping status

Note: there were some missing data on gender (unweighted *n* = 206; *n* = 52 who did not respond and *n* = 154 who identified as non-binary); these cases were excluded from the adjusted models

Exclusive smokers in general were less likely than dual users to think e-cigarettes were less harmful than cigarettes. Among exclusive smokers, age differences in harm perceptions were similar to those observed for all smokers. For example, 30.3% of older adults were uncertain compared with 14.6% of those who were younger or middle-aged (OR_adj_ = 2.58 [2.37–2.82]).

Harm perceptions deteriorated across the study period in both age groups (Additional File 1: Fig. S1). Sensitivity analyses restricted to data collected between April 2022 and April 2024 showed that although the proportion perceiving e-cigarettes as less harmful was lower than in our primary analyses (21.0% and 25.8% among older and younger and middle-aged adults, respectively), the pattern of age differences was similar (Additional File 1: Table S1).

### Real-world effectiveness of e-cigarettes for smoking cessation

Among past-year smokers who made at least one quit attempt in the past year (unweighted *n* = 1140 older adults, *n* = 10,563 younger and middle-aged adults), e-cigarettes appeared effective for short-term (< 1 year) smoking cessation. After adjustment for covariates, age-stratified analyses suggested use of e-cigarettes was associated with 95% higher odds of self-reported continuous abstinence from the start of the most recent quit attempt to the time of the survey, compared with non-use, among younger and middle-aged adults (OR_adj_ = 1.95 [1.72–2.21]) and 50% higher odds among older adults (OR_adj_ = 1.50 [0.96–2.34]). The 95%CI was wider for the older adult effect estimate, on account of the much smaller sample size, and included the possibility of no difference in quit success between those who did and did not use e-cigarettes.

The interaction between the use of e-cigarettes and the age group on success in stopping smoking was not statistically significant (interaction OR_adj_ = 0.78 [0.51–1.21]). This result was robust to sensitivity analyses (OR_adj_ = 0.78 [0.50–1.22] when we adjusted for education rather than occupational social grade and OR_adj_ = 0.79 [0.51–1.23] when we also adjusted for past-year receipt of GP advice on smoking). Bayes factors indicated the data supported no difference in effectiveness between age groups as being more likely than an association of OR ~ 1.5 (i.e. e-cigarettes being more effective for older people; BF = 0.26) but were insensitive to e-cigarettes being less effective for older people (BF = 1.30).

## Discussion

This study explored differences between older (≥ 65 years) and younger and middle-aged adults (18–64 years) in England in trends in tobacco smoking and vaping, harm perceptions of e-cigarettes relative to cigarettes, and the real-world effectiveness of e-cigarettes for smoking cessation.

We found that trends in smoking and vaping prevalence among older adults over the past decade differed from those observed in the rest of the adult population. Between April 2014 and April 2024, tobacco smoking prevalence remained relatively unchanged among older adults and the proportion who vaped increased. However, among younger and middle-aged adults, there was a clear decline in smoking (although prevalence remained higher than among older adults) and a larger increase in vaping. These patterns are consistent with findings from other national surveys in England documenting smaller declines in smoking and smaller increases in vaping among older adults [8, 34]. The extent of the decline in smoking we observed in younger and middle-aged adults was lower, and absolute smoking prevalence among both groups was higher than in other national surveys [8]. This is likely because our definition of current smoking included all combustible tobacco products, not just cigarettes; the proportion of adults in England who do not smoke cigarettes but smoke some other form of tobacco has increased since 2020 [15]. While the reasons for this increase in non-cigarette tobacco smoking are unclear, it could be linked to the UK’s 2020 menthol ban, which covered cigarettes but not other combustible tobacco products (e.g. cigarillos), or a shift to online purchasing during the pandemic exposing people to more non-cigarette products [15].

There were different patterns in age-specific smoking and vaping trends by socioeconomic position. Any evidence of a decline in smoking among older adults was limited to the more advantaged social grades and absent in less advantaged social grades. This echoes recent US data showing less favourable trends in smoking among older adults on lower compared with higher income levels [14]. By contrast, declines in smoking among younger adults were more pronounced in the less advantaged social grades. This suggests initiatives aiming to reduce inequalities in smoking (a priority for tobacco control in England) to date may have been less successful in reaching or encouraging quitting among older adults. Although this possibility is not easily reconciled with the age differences in vaping observed by different social grades: increases in vaping prevalence among younger and middle-aged adults were observed across social grades, but only occurred among older adults from less advantaged social grades. The reasons for this are unclear. Qualitative research would be useful to better understand the reasons for these different patterns.

Older adults remained less likely than younger and middle-aged adults to use e-cigarettes to support attempts to quit smoking (although there may have been a greater increase over time among older compared with younger and middle-aged adults). It is possible differences in harm perceptions may have contributed to the lower absolute prevalence among older adults. Older smokers reported greater uncertainty than younger and middle-aged smokers about the harms of e-cigarettes compared with cigarettes. A similar pattern has been observed previously in a survey of US adults [35], although the absolute misperceptions appear higher in the USA. Research in the UK has found older smokers tend to have poorer knowledge about nicotine than younger smokers, more commonly misattributing risks of smoking (e.g. cancer) to nicotine [36]. There may be value in targeting older smokers with evidence-based information about the risks of vaping in order to enable informed decision-making. Among those who both smoked and vaped, older adults were more likely than younger adults to perceive e-cigarettes as less harmful than cigarettes. This could imply accurate perceptions of the relative harms are more important in encouraging older than younger and middle-aged smokers to take up vaping.

E-cigarettes appeared to be effective for helping older adults to stop smoking, but our data were inconclusive as to whether effectiveness was lower than for younger and middle-aged adults. After adjusting for relevant confounders, we found the odds of quitting were around 50% higher for older adults who used an e-cigarette than those who did not and around 95% higher for younger and middle-aged adults who did so. The point estimate for the interaction term was OR = 0.78, suggesting e-cigarettes might be less effective for older adults, but the 95% CI included the possibility of no difference or e-cigarettes being more effective (upper CI = 1.21). More data are needed to draw firm conclusions. If effectiveness *is* found to be lower among older adults, then we will also need more research to understand why. Possible explanations might include differences in the products used (e.g. nicotine strengths), how often they are used, and for how long. We did not have a sufficient sample size to explore this. However, in the absence of clear evidence that effectiveness differs, it can be assumed that e-cigarettes remain a good option to support older adults to stop smoking—as evidenced by a growing number of high-quality randomised controlled trials [10] and observational individual- and population-level studies [13, 21, 28, 37–40].

### Implications

Collectively, these findings suggest there is an opportunity to boost smoking cessation among older adults by increasing their use of e-cigarettes to support quit attempts, which could help to reduce inequalities in smoking [41]. Further research is needed to better understand why older adults are less likely to use e-cigarettes and how e-cigarettes could be promoted successfully to encourage older adults to use them to quit smoking, and the extent to which this could have positive equity impacts.

England’s approach to vaping is distinct from many other countries in which vaping for smoking cessation is not supported by the government or most major medical and public health organisations. Examining age-specific trends in smoking and vaping can offer some insight into the impact of England’s approach to vaping position on smoking rates. Recent trend analyses suggest declines in smoking since 2021 have been greatest among age groups with the largest increases in vaping [42]. However, a trade-off is increased uptake of vaping among young people who have never regularly smoked tobacco (although some of this will likely reflect some people being diverted away from ever taking up smoking) [43]. Our present results show a similar pattern over a longer period: a greater decline in smoking and a greater increase in vaping among younger and middle-aged adults compared with older adults, but an increase in vaping among younger and middle-aged adults who had never regularly smoked. Policies should aim to strike the right balance between making vaping attractive and accessible to smokers to support quitting while minimising uptake by people who would not otherwise smoke. Failing to promote e-cigarettes as a smoking cessation aid is out of line with evidence that this is one of the most effective methods of quitting [10, 20] and risks perpetuating misperceptions of vaping and keeping people smoking for longer.

### Strengths and limitations

Strengths of the study included the large, representative sample; the detailed assessment of smoking, vaping and quit attempts; and the monthly data collection. There were also several limitations.

There was a change in methodology (from face-to-face to telephone interviews) at the start of the COVID-19 pandemic. However, the two modes show good comparability [24] and our results show no indication that there was a substantial mode effect on any of our outcomes of interest (see unmodelled data points in Figs. 1, 2, and 3).

Sample sizes were relatively small for some analyses, meaning there may be differences between older and younger and middle-aged adults that we were unable to detect. Indeed, Bayes factors indicated that our analysis of differences in the real-world effectiveness of e-cigarettes was insensitive to distinguish between no difference and effectiveness being lower among older adults. It may be useful to revisit this analysis when more data are available.

Although our real-world effectiveness analyses adjusted for a range of relevant confounders, there may be residual confounding by unmeasured factors (e.g. frequency and duration of e-cigarette use). In addition, there was no standardised definition of abstinence: participants who reported continuous abstinence from the start of the most recent quit attempt up to the time of the survey, which could include periods of abstinence of any duration up to a year, were considered to have successfully quit. However, we did adjust for the time since the quit attempt started in the analysis so while this means the outcome will be a mixture of different durations of abstinence, it is accounted for in terms of success across comparisons.

Our definition of older adults (≥ 65 years) encompassed a wide range of ages and there are likely to be differences within this group that were not captured here (including possible differences by social grade). Further, self-selection (such as healthy survivor effects) impacts older age groups more than others such that the typical smoker (or vaper) aged 65 or above is likely quite different from one below this age. There may also be cohort effects that we did not account for (e.g. differences in smoking rates at equivalent ages or in the distribution of the ≥ 65 years group over the decade). While it is possible that the interaction terms between time and age in our models partly capture any such effects, further research could explore the consequences of such biases in more detail (e.g. using age-period-cohort or propensity score matching approaches).

Our comparator group of younger and middle-aged adults also included a wide age range (18–64 years). We chose to analyse these participants as a single group because our focus was on older adults, rather than differences across the age span. However, other evidence shows there is variation in our outcomes of interest within this group [8, 18], which may have led us to underestimate some age-related differences (e.g. differences in vaping prevalence would have been more pronounced if we had compared older adults with young adults specifically).

Finally, our results are specific to England and may not generalise to other countries with different approaches to tobacco control or vaping. In addition, people living in older adult care homes were not included in the sample, so results may not generalise more broadly within England. The current study has high external validity because it reflects how people smoke and vape in the real-world outside of clinical trial settings. However, there is less internal validity than for RCTs because people self-selected into using e-cigarettes for smoking cessation, and despite our adjustment for important confounders, there likely remain important sources of unmeasured confounding.

## Conclusions

Our data show that tobacco smoking prevalence has fallen more slowly over the past decade among older adults than the rest of the adult population in England. Older adults are more unsure about the relative harms of e-cigarettes and less likely to use them to support attempts to quit smoking, despite evidence suggesting they are effective for smoking cessation.

## Supplementary Information

Additional File 1: Fig. S1, Table S1. FigS1 – Harm perceptions of e-cigarettes compared with cigarettes among current smokers across the study period TableS1 – Unadjusted and adjusted associations of age with harm perceptions of e-cigarettes relative to cigarettes, among current smokers – sensitivity analysis (April 2022 – April 2024)

## Data Availability

Data are available on Open Science Framework (https://osf.io/yg7nr/).

## Abbreviation

NRT: Nicotine replacement therapy
