# Supplementary material for: Use, perceptions, and effectiveness of e-cigarettes for smoking cessation among older adults in England: a population study, 2014–2024
Source: BMC Med. 2024 Oct 31;22:500. doi: 10.1186/s12916-024-03728-x (PMC11526569; doi:10.1186/s12916-024-03728-x)
Supplement: Supplementary file 1 — Additional File 1: Fig. S1, Table S1. FigS1 – Harm perceptions of e-cigarettes compared with cigarettes among current smokers across the study period TableS1 – Unadjusted and adjusted associations of age with harm perceptions of e-cigarettes relative to cigarettes, among current smokers – sensitivity analysis (April 2022 – April 2024) [file 12916_2024_3728_MOESM1_ESM.pdf]

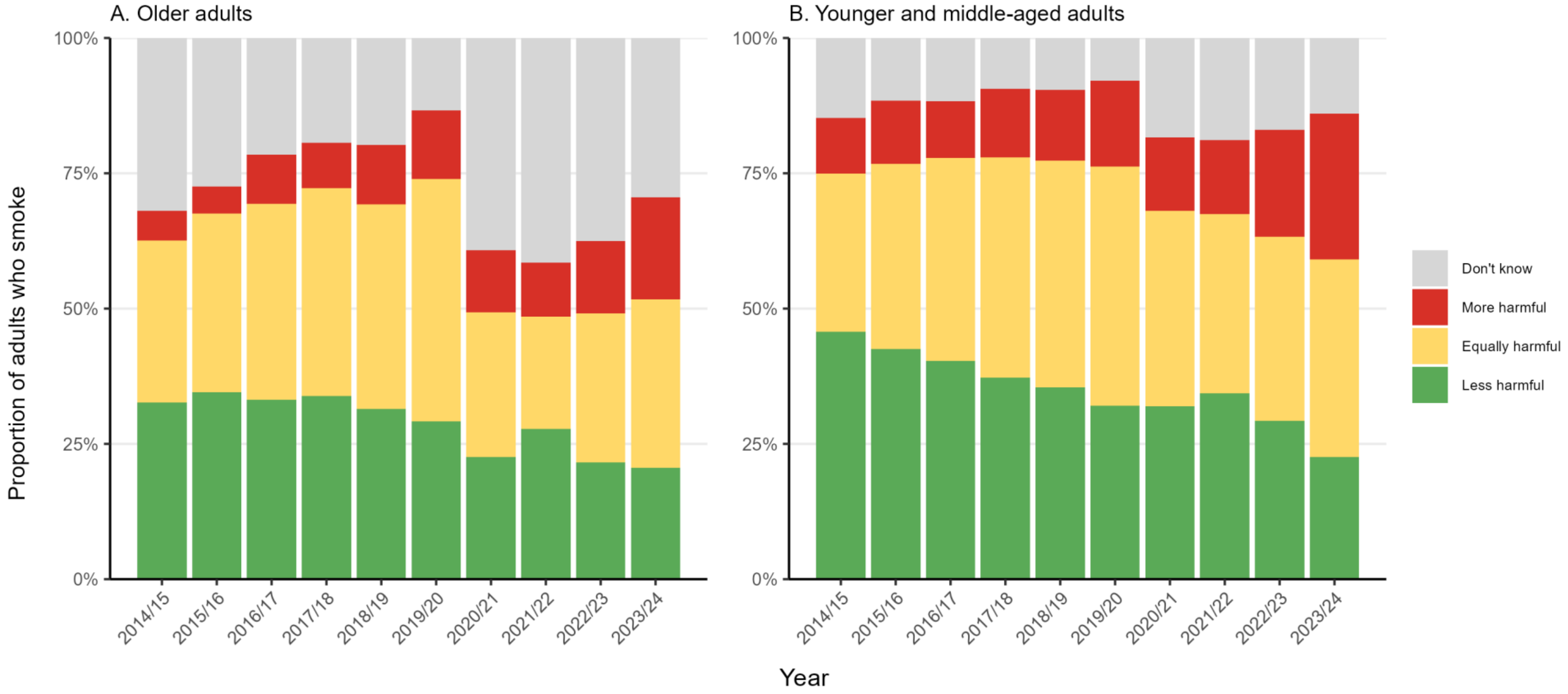


**Figure S1. Harm perceptions of e-cigarettes compared with cigarettes among current smokers across the study period.** Data shown are unmodelled proportions of older smokers and middle-aged and younger smokers who perceived e-cigarettes to be less harmful, equally harmful, more harmful than cigarettes, or who did not know. Survey year was categorised in 12-month periods from April to March (e.g., 2013/14 = April 2013 to March 2014).

**Table S1.** Unadjusted and adjusted associations of age with harm perceptions of e-cigarettes relative to cigarettes, among current smokers – sensitivity analysis (April 2022 – April 2024)

| | Prevalence, % [95% CI] | | | |
|---|---|---|---|---|
| | **Middle-aged/ younger adults** | **Older adults** | **OR [95% CI]** | **$OR_{adj}$ [95% CI]** |
| **All smokers (*n*=6,164)** | | | | |
| Less harmful | 25.8 [24.5–27.1] | 21.0 [18.2–23.9] | 0.77 [0.64–0.92] | 1.08 [0.89–1.31] |
| Equally harmful | 35.3 [33.9–36.7] | 29.5 [26.1–32.8] | 0.77 [0.64–0.91] | 0.73 [0.61–0.86] |
| More harmful | 23.5 [22.3–24.8] | 16.3 [13.6–18.9] | 0.63 [0.51–0.78] | 0.51 [0.41–0.63] |
| Don't know | 15.4 [14.3–16.5] | 33.2 [29.8–36.7] | 2.74 [2.30–3.26] | 2.53 [2.12–3.02] |
| **Exclusive smokers (*n*=4,351)** | | | | |
| Less harmful | 18.2 [16.9–19.6] | 16.2 [13.4–18.9] | 0.86 [0.69–1.08] | 0.93 [0.74–1.16] |
| Equally harmful | 35.6 [33.9–37.3] | 30.3 [26.7–33.9] | 0.79 [0.65–0.95] | 0.76 [0.63–0.92] |
| More harmful | 28.4 [26.8–30.0] | 17.8 [14.8–20.8] | 0.55 [0.44–0.68] | 0.51 [0.41–0.64] |
| Don't know | 17.8 [16.4–19.1] | 35.7 [31.9–39.5] | 2.57 [2.13–3.11] | 2.67 [2.21–3.23] |
| **Dual users (*n*=1,813)** | | | | |
| Less harmful | 41.2 [38.7–43.8] | 55.8 [45.3–66.3] | 1.80 [1.17–2.77] | 1.90 [1.23–2.94] |
| Equally harmful | 34.7 [32.2–37.2] | 23.2 [14.0–32.5] | 0.57 [0.34–0.96] | 0.53 [0.31–0.90] |
| More harmful | 13.5 [11.8–15.3] | 5.3 [0.6–10.0] | 0.36 [0.14–0.91] | 0.37 [0.14–0.93] |
| Don't know | 10.6 [9.0–12.2] | 15.7 [8.1–23.3] | 1.57 [0.87–2.84] | 1.58 [0.87–2.84] |

CI, confidence interval.
OR, odds ratio for harm perceptions among older adults (≥65 years) compared with younger and middle-aged adults (18-64 years; reference group).
$OR_{adj}$, odds ratio adjusted for gender, occupational social grade, and (for analyses among all smokers) vaping status.
Note: there were some missing data on gender (unweighted *n*=133; *n*=52 who did not respond and *n*=81 who identified as non-binary); these cases were excluded from the adjusted models.
